# Hyaluronic Acid and Its Synthases—Current Knowledge

**DOI:** 10.3390/ijms26157028

**Published:** 2025-07-22

**Authors:** Klaudia Palenčárová, Romana Köszagová, Jozef Nahálka

**Affiliations:** 1 Institute of Chemistry, Centre for Glycomics, Slovak Academy of Sciences, Dubravska Cesta 9, SK-84538 Bratislava, Slovakia; chemktal@savba.sk (K.P.); chemrore@savba.sk (R.K.); 2 Institute of Chemistry, Centre of Excellence for White-Green Biotechnology, Slovak Academy of Sciences, Trieda Andreja Hlinku 2, SK-94976 Nitra, Slovakia

**Keywords:** hyaluronic acid, hyaluronic acid synthase, production, in vitro synthesis

## Abstract

Hyaluronic acid (HA) is a linear heteropolysaccharide that naturally occurs in vertebrates. Thanks to its unique physico-chemical properties, it is involved in many key processes in living organisms. These biological activities provide the basis for its broad applications in cosmetics, medicine, and the food industry. The molecular weight of HA might vary significantly, as it can be less than 10 kDa or reach more than 6000 kDa. There is a strong correlation between variations in its molecular weight and bioactivities, as well as with various pathological processes. Consequently, monodispersity is a crucial requirement for HA production, together with purity and safety. Common industrial approaches, such as extraction from animal sources and microbial fermentation, have limits in fulfilling these requests. Research and protein engineering with hyaluronic acid synthases can provide a strong tool for the production of monodisperse HA. One-pot multi-enzyme reactions that include in situ nucleotide phosphate regeneration systems might represent the future of HA production. In this review, we explore the current knowledge about HA, its production, hyaluronic synthases, the most recent stage of in vitro enzymatic synthesis research, and one-pot approaches.

## 1. Introduction

Polysaccharides are abundantly occurring macromolecular polymers in nature. Unlike proteins and polynucleotides, polysaccharides are characterized by a large variety of architectures. Their molecules can be linear, branched, or networked, and monomeric units within the molecule can be connected by various types of linkages. This structural variability predisposes them to fulfilling numerous roles in living organisms [1,2].

**Figure 1:**
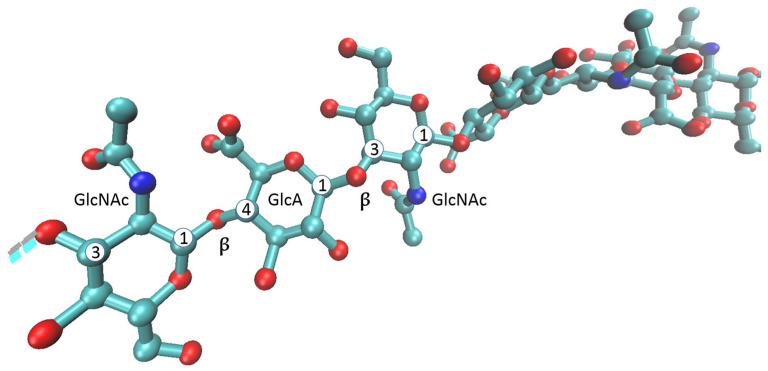
Hyaluronic acid structure: [-3-GlcNAc-1-β-4-GlcA-1-β-]*_n_*. GlcA—D-glucuronic acid, GlcNAc—N-acetyl-D-glucosamine. The coordinates were downloaded from the Protein Data Bank; 8SMN is the corresponding PDB code, and Visual Molecular Dynamics (VMD 1.9.3) was used for visualization.

Hyaluronic acid (HA) is a heteropolysaccharide with a linear molecule, consisting of repeating disaccharide units made up of N-acetyl-D-glucosamine (GlcNAc) and D-glucuronic acid (GlcA) [-3-GlcNAc-1-β-4-GlcA-1-β-]*_n_* (Figure 1). It was first isolated in 1934 by Karl Meyer and John Palmer from the bovine eye vitreous body. Its name is a conjugation of hyaloid (vitreous body) and uronic acid [3]. It is ubiquitously present in vertebrates, but it has also been identified in some mollusks, fungi, bacteria, and algal viruses. In microorganisms, the production of HA is a virulence factor. In vertebrates, including humans, HA fulfills a variety of functions. It is a major compound of the extracellular matrix, eye vitreous humor, and cartilage joints and, as a signaling molecule, it plays a crucial role in an array of physiological and pathological processes, such as fertilization, embryogenesis, inflammation, wound healing, angiogenesis, and even carcinogenesis [4]. The molecular weight (MW) of HA can vary greatly: very-high-molecular-weight (vHMW) HA (>6000 kDa), high-molecular-weight (HMW) HA (>1000 kDa), low-molecular-weight (LMW) HA (10–1000 kDa), and HA oligosaccharides (o-HAs) (<10 kDa). HA’s MW is a key determinant of its biological functions, and might even be opposite [5,6]. The biological functions of HA also determine its broad range of applications in cosmetics, the food industry, and medicine, e.g., wound healing, ophthalmology, pneumology, orthopedics, drug nanocarriers, and tissue engineering scaffolds [6,7,8].

The global HA market size was estimated at USD 11.14 billion in 2024 and predicted to increase from USD 11.81 billion in 2025 to approximately USD 15.84 billion in 2030 [9]. For this reason, there is a need for efficient and cheap large-scale production. At present, traditional extraction from animal tissues is often substituted with microbial fermentation. However, increasing requirements for safety, purity, and the low dispersity of the final product—especially regarding the dependence of HA’s properties on its MW—have aroused interest in the enzymatic synthesis of HA in vitro. The protein engineering of key enzymes—hyaluronic acid synthases (HASs)—is a promising tool for HA synthesis with restricted dispersity. HASs catalyze the polymerization of the monomeric units UDP-GlcA and UDP-GlcNAc, which are expensive substrates. Coupling HASs with other enzymes into one-pot multi-enzyme reactions enables the use of cheap primary substrates, crucially regenerating nucleotide phosphates [10,11].

In this review, we summarize the current research on HA, including methods of production, progress in the study of HASs, and enzymatic synthesis in vitro, including one-pot approaches and nucleotide phosphate regeneration systems.

## 2. Structure and Properties of HA

HA belongs to the glycosaminoglycan group of heteropolysaccharides. Unlike other glycosaminoglycans (i.e., chondroitin sulfate, dermatan sulfate, keratin sulfate, heparin sulfate, and heparin), HA is not sulfated and is not synthesized by Golgi enzymes; thus, it is not covalently bound to the core protein [4,11]. Synthesis by HASs takes place on the cytoplasmic surface of the plasma membrane, and the HA is usually directly extruded through plasma membranes into the extracellular space [3]. HA also differs in size, as it can reach up to 10^4^ kDa, whereas other glycosaminoglycans range from 15 to 20 kDa [4]. It is an unbranched linear polysaccharide composed of repeating disaccharide units, which consist of GlcNAc and GlcA linked by alternating β-1,4 and β-1,3 glycosidic bonds (Figure 1) [7,12]. In the β-conformation, hydroxyl, carboxyl, acetamido, and anomer carbons are in sterically favorable equatorial positions, making polysaccharide chains very energy-stable. The hydrogens occupy less favorable axial positions. In this rigid conformation, with limited rotation around the glycosidic bonds of the HA backbone, hydrophobic groups alternate with polar groups linked by intra- and inter-molecular hydrogen bonds [4]. At physiological pH, each carboxyl group carries an anionic charge, which can interact with mobile cations and form salts called hyaluronan or hyaluronate [4,13]. HA is highly hydrophilic due to the many carboxyl and hydroxyl groups in its molecule, allowing it to hold approximately 1000 times its weight in water [5]. Water molecules stabilize the secondary structure of HA, a two-fold single helix, as they link HA carboxyl and acetamido groups with hydrogen bonds. These two-fold helices form in aqueous solution duplexes, i.e., a β-sheet tertiary structure, formed by hydrophobic interactions between hydrophobic regions and intermolecular hydrogen bonding between antiparallel HA chains, enabling the aggregation of polymeric chains [4,12]. The establishment of polymeric networks is dependent on HA’s molecular weight and concentration, as an increase in both parameters results in a strengthened network, as well as increased viscosity and viscoelasticity. HA solutions behave as non-Newtonian fluids with viscoelastic and shear-thinning (pseudoplastic) characteristics. The rheology of HA is strongly influenced by the ionic strength of the solution, pH, and temperature. At pH values below 4 or above 11, hydrolytic degradation of HA occurs, resulting in disintegration of the polymer network and reduced viscosity [4,13]. Under acidic conditions, the proton attacks the GlcA unit, while the alkaline hydroxide ion attacks the GlcNAc [14]. The polymer network’s breakdown and shift from a gel to a liquid is transient and reversible [4]. The unique properties of HA are crucial for its physiological roles and its applications in various industrial fields [4,12].

## 3. Biology of HA

HA is abundantly distributed in the human body as well as in other vertebrates. Additionally, it can be found in some mollusks, yeasts, bacteria, and algal viruses. To date, it has not been identified in fungi, plants, or insects [4]. In the human body, HA acts as a passive structure on the basis of its physico-chemical properties, or as a signaling molecule by interacting with its binding proteins.

As a passive structure, HMW HA is a major component of the extracellular matrix of connective tissue and forms a pericellular coating around cells. HA is also an important component of the skin in both the dermis and epidermis. Due to its unique hygroscopic properties, it hydrates the skin and, together with collagen and elastin fibers, gives skin tissue its characteristic physical properties and thus helps it to fulfill its natural role [15]. HA also works as a lubricant and space filler in synovial fluids, reducing fractions and abrasions and absorbing pressure between articular cartilage. Moreover, it participates in the transport of nutrients to cartilage. Inflammation processes often decrease pH and increase reactive oxygen species (ROS), decreasing the thickness and viscosity of HA in synovial fluid, thus promoting disorders such as arthritis [16,17]. HA is also part of the vitreous body where together with collagen and water, it is responsible for the typical jelly-like composition of the eye bulb and moisturization. HA also acts as an eye moisturizer, as an element of tear fluid [18], and plays a structural role in the umbilical cord as a major component of Wharton’s jelly, together with chondroitin sulfate [4].

**Figure 2:**
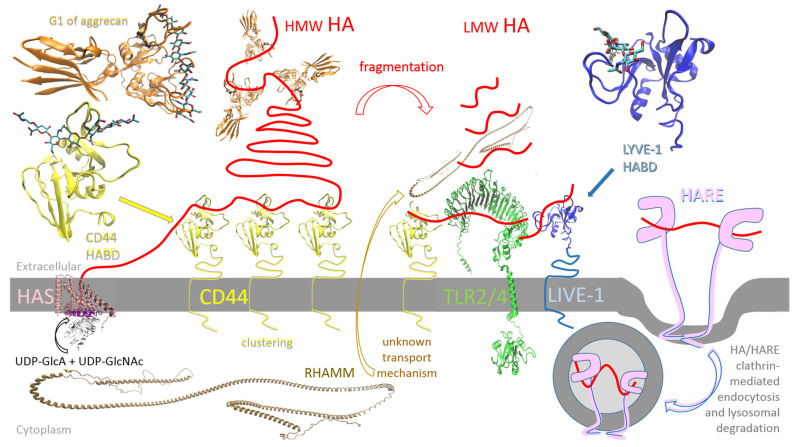
Biology of hyaluronic acid (HA). HA (red) is synthesized and exported via hyaluronic acid synthase (HAS) into a high-molecular-weight polymer (HMW HA), which interacts with cell receptors and proteoglycans/hyaladherins. CD44 (yellow) is the primary cell surface receptor for HA. Aggrecan is a widely distributed chondroitin sulfate proteoglycan; its G1 region (orange) interacts with HA. HMW HA-CD44 interactions cluster more CD44. HA fragmentation initiates the expression and export of the CD44 coreceptor RHAMM (brown). Unclustered CD44, its unclustered homolog LIVE-1 (blue), TLR2 (green), TLR4, and the other receptors bind LMW HA and provide HA-fragmentation signaling. The HARE receptor (pink) binds to HA and mediates clearance from biological fluids via clathrin-mediated endocytosis. These coordinates were downloaded from the Protein Data Bank and AlphaFold Protein Structure Database; 2JCR (CD44, *Mus musculus*), 9DFF (aggrecan, *Homo sapiens*), 8OXD (LYVE-1, *Homo sapiens*), 8SMN (HAS1, *Xenopus laevis*), and O60603 (AF-TLR2, *Homo sapiens*) are the corresponding codes.

HA also functions as a signaling molecule and coregulates numerous physiological and pathological processes, including reproduction, embryonic development, inflammation, tissue regeneration, cell migration, proliferation, angiogenesis, and cancer [4,19]. These functions are highly dependent on HA’s MW, location, and cell-specific factors. HMW HA cannot penetrate cells due to its huge size. LMW HA and o-HAs can enter the cell through the membrane and interact with intracellular signaling molecules, e.g., transcription factors and intracellular kinases. Short o-HAs consisting of 4–8 monosaccharides are even distributed in the cell nucleus [6]. HMW and LMW HA can even act antagonistically. Extracellular HMW HA is anti-angiogenic, as it inhibits the growth of endothelial cells and is useful in inflammatory processes, tissue injury and repair, wound healing, and immunosuppression. Under specific environmental and pathological conditions, HMW HA can be degraded into LMW HA, which has the opposite effect (Figure 2). This induces pro-inflammatory and pro-angiogenic processes and can even promote extracellular matrix remodeling and tumor progression. Anti- and pro-inflammatory activity has also been observed in o-HAs, depending on the cell type and disease [4].

HA interacts with HA-binding proteoglycans, i.e., hyaladherins, such as aggrecan in cartilage; neurocan and brevican in the central nervous system; and versican in soft tissue or receptors on the cell surface [19]. HA can interact with its receptors on the same cell (autocrine manner) or with receptors on adjacent cells (paracrine manner) to facilitate intracellular signaling; in the case of HMW HA, a single chain can interact with several hyaladherins at the same time [4]. The most important cell receptors for HA are cluster determinant protein 44 (CD44), receptor for hyaluronan-mediated motility (RHAMM), lymphatic vessel endothelial hyaluronan receptor (LYVE-1), hyaluronan receptor for endocytosis (HARE), toll-like receptors (TLRs), and layilin (Figure 2) [6,19]. CD44 is the primary cell surface receptor for HA, and the interaction between the two is part of many physiological events, including cell–cell and cell–substrate adhesion, cell migration, cell proliferation, and hyaluronan uptake and degradation, as well as pathological processes such as cancer progression. CD44 is constitutively expressed in most tissues, but it is only present in alternatively spliced isoforms in a cell-type-specific and context-specific manner. These isoforms may differ in glycosylation patterns and interreceptor interactions. CD44 acts through linking the cortical actin cytoskeleton with the pericellular HA matrix and thus maintains a protective and homeostatic cellular microenvironment. HMW HA–CD44 interactions can cluster more CD44 (Figure 2), whereas HA oligosaccharides cannot. Interestingly, HA oligosaccharides can even reduce the clustering caused by HMW HA. HA fragmentation caused by tissue injury and cellular stress leads to the unconventional export of the CD44 coreceptor RHAMM (Figure 2), which alters the homeostatic organization of CD44/HA and is associated with growth factor receptors. These events trigger cellular responses, such as cell motility, that participate in tissue repair and disease processes, including cancer progression [20]. RHAMM is also present in various isoforms and is localized in microtubules, the centrosome, and the cytosol. It influences microtubule dynamics, centrosomes, and the mitotic spindle, as it is required for the transition through the G2M phase of the cell cycle. It is also part of transcriptional complexes in cell nuclei [19]. This is consistent with findings about the intracellular location of HA and its association with microtubules, RHAMM, and mitotic spindle [21]. However, we do not know if these functions are associated with extracellular HA-binding. RHAMM is transported to the cell surface as a response to injury or stress, as noted, but the mechanism of this transport is not fully understood [20].

LYVE-1 is found on the endothelial cells of lymph vessels and some types of macrophages. It recognizes HA from glycocalyx, e.g., dendritic and vascular smooth muscle cells, and thus regulates leucocyte trafficking and contributes to macrophage function. LYVE-1 on the lymph endothelium uptakes HMW and intermediate HA from tissues to lymph and induces lymphangiogenesis after interaction with LMW HA (Figure 2) [19].

HARE is responsible for the binding and endocytosis of HA and its ligands as a part of normal turnover process of HA from the circulatory system (Figure 2). After internalization, HA degrades in the lysosome. Layilin is highly upregulated in certain types of T cells during disease processes; it binds HA and interacts with cytoskeletal proteins talin-1 and merlin and, so, likely participates in cell adhesion [19]. TLRs are implemented in the immune response against pathogens. The TLR signal cascade can be downregulated by the CD44 signal cascade, which prevents excessive inflammation. HMW HA interacts with TLR and can simultaneously interact with CD44, which is crucial in the inflammatory process. LMW HA binds only to TLRs, enhancing the inflammation process [19,22].

HA has been identified in some mollusks, where it probably regulates cell functions such as proliferation and migration in embryonic development, processes following injury, immune reactions, and inflammation [23,24]. In single-cell organisms, HA is produced by certain pathogenic bacteria (*Streptococci*, *Pasteurella multocida*, *Bacillus anthracis*, *Haemophillus influenzae*) and yeasts (*Cryptococcus neoformans*). It is integrated in their capsula, where it facilitates adhesion or colonization and is a part of the organism’s strategy to escape the host immune system [25,26,27]. Interestingly, *Chlorella* cells infected with PBCV-1 (*Paramecium bursaria Chlorella* virus type 1) have been observed to produce HA. HA’s role in the virus life cycle is not yet clearly understood, but it has been suggested to prevent secondary virus infection, stabilize host cells to increase virus burst size, and/or facilitate interactions with other potential host organisms [28,29].

## 4. Production of HA

### 4.1. Extraction from Animal Sources

Extraction from animal sources is a traditional way to obtain HA and has been used commercially since the early 1940s [30]. Given that HA is naturally produced in vertebrates, it can be sourced from various parts of terrestrial and marine animals, e.g., cartilage, head, eyes, fins, and skin. As such, it is basically considered as a cheap waste by-product of animal production. Rooster comb, vitreous humor, umbilical cord, and synovial fluid are the most widespread animal sources for the commercial extraction of HMW HA. The extraction process generally includes tissue hydrolysis, protein removal, and subsequent HA purification. There are various drawbacks to this method. HA obtained through extraction is polydisperse; i.e., it contains a large number of molecules of variable chain lengths. This might be a problem for commercial applications, as HA properties are chain-length-dependent [31,32]. Isolation can be difficult due to the formation of stable complexes of HA and proteoglycans [33], and the final product can be contaminated with other structurally similar glycosaminoglycans, which might be difficult to separate from HA [32]. There are even safety concerns, such as a risk of infection by animal viruses and prior impurities [14,33].

### 4.2. Fermentation

Research on HA production via bacterial fermentation began in the 1980s, which was first focused only on natural producers. The most extensively used producer is *Streptococcus equi* subsp. *zooepidemicus*, as it continuously secretes HA into the extracellular space with minimal byproducts and has a short fermentation cycle with relatively low production costs [30]. Various techniques, including site-directed mutagenesis, genome shuffling, artificial transcription factor engineering, random mutagenesis, target gene overexpression, laboratory evolution, high-throughput screening, and gene engineering, have been applied to increase substrate conversion efficiency, minimize by-product production, and increase stress tolerance in native strains [34]. As noted, natural producers are pathogens that synthesize HA as a part of their virulence, raising concerns about the safety of the target product in biomedical applications [30,33]. The development of gene engineering has opened up the possibility of producing HA using non-pathogenic organisms. Genes from natural producers are transformed into host organisms to reconstruct the HA biosynthetic pathway. Recombinant microorganisms for HA biosynthesis can be found among Gram-positive bacteria (e.g., *Lactobacillus lactis*, *Bacillus subtilis*, and *Corynebacterium glutamicum*), Gram-negative bacteria (e.g., *Escherichia coli* and *Agrobacterium*), and fungi (e.g., *Saccharomyces cerevisiae* and *Pichia pastoris*). However, the product yields and MW achieved by natural producers always surpass those from recombinant hosts due to plasmid instability. Nonetheless, they are considered a safe and cost-effective alternative because they do not require safety equipment for purification from toxins [33].

Fermentation-based HA production has been sidelined in favor of the extraction method because it involves simpler processing, is free from instabilities, and is not influenced by seasonal variations [34]. There are several issues in industrial HA production via fermentation. HA synthesis is inhibited as a result of the accumulation of lactic acid, which is a coproduct of enzyme synthesis; furthermore, HA itself increases substrate viscosity, decreasing mixture saturation with oxygen [33]. Another challenge is the reduction in dispersity; to some extent, this might be influenced by precisely controlling the cultivation conditions, such as temperature, pH, agitation, dissolved oxygen, and substrate concentrations [5,34]. To produce HMW HA, it is necessary to increase dissolved oxygen levels and agitation speed. However, high oxygen levels might increase ROS, which degrade HA and decrease its MW, although this problem might be solved through the addition of salicylic acid. Under anaerobic conditions, the expression of the key enzyme HAS and its activity are inhibited [35]. Other options for increasing HA’s MW include enzyme engineering strategies and the regulation of genes related to HA synthesis, as the intracellular concentration and ratio of substrate molecules impact the yield and MW of HA [34,35]. A sucrose-inducible expression system for regulation of the *hasE* gene has been shown to enable the production of HA with specific MWs through a one-step fermentation process. *hasE* encodes phosphoglucoisomerase in *S. equi* subsp. *zooepidemicus* and is a key factor in balancing HA precursors; the disruption of this balance results in the production of LMW HA [36]. However, these strategies are not efficient in producing LMW HA and o-HAs. Therefore, the most common approach involves the enzymatic degradation of HA using hyaluronidase; this technique provides good reproducibility, controllable degradation, and mild reactions [35]. Different LMW HAs were produced using *B. subtilis* by altering the cultivation temperature, while leech hyaluronidase was expressed on a temperature-sensitive plasmid [37]. Zhong et al. achieved the production of HA with tailored MWs in *B. amyloliquefaciens* by expressing hyaluronidase taken from *Streptomyces thermolilacinus* [38]. The addition of hyaluronidase to the process of fermentation can reduce viscosity and improve production, as found in the two-stage semi-continuous fermentation of *S. equi* subsp. *zooepidemicus* [39]. Di et al. achieved reduced MW and increased HA titers in *S. equi* subsp. *zooepidemicus* by overexpressing the cysteine transporter *fliY1*. A significant reduction in HA MW in the same producer was also observed after knockout of the lactate dehydrogenase gene *ldh* [40]. However, HA produced via fermentation must be extracted and purified from the producing cells and fermentation broths. This usually requires (i) precipitation to remove proteins, lipids, and nucleic acids; (ii) filtration to remove remaining insoluble impurities and improve purity; and (iii) adsorption by passing the solution through an anion-exchange chromatography column, where only HA is bound and other components are washed out. HA is then eluted using a salt solution [8].

### 4.3. In Vitro Synthesis

HA can be synthesized using chemical, physical, chemoenzymatic, or enzymatic methods. The chemical approach includes click chemistry reactions, where the main problem is product contamination caused by residual chemical cross-linking agents. Self-oxidation by catechol groups and UV or visible light irradiation enables toxic chemical cross-linking agents to be avoided [41,42]. LMW HA and HA oligosaccharides can be prepared through the degradation of HMW HA. Except for the enzymatic approach using hyaluronidase, this can be accomplished using acidic or alkaline hydrolysis, ROS, or a combined physical and chemical method, such as ultrasound combined with hydrogen peroxide and copper ion [14]. Physical methods include ultrasound, ozone, electron beam, heat treatment, and γ-rays [43,44]. Irradiation with γ-rays reduces the MW of HA without altering the structure of the polymer [43], unlike, e.g., hyaluronidase cleavage or pH-driven hydrolysis, which lead to the formation of unsaturated bonds [45]. Degradation is random and difficult to control; thus, the result is a polydisperse mixture that must be fractionated to obtain homogenous HA oligosaccharides [14]. However, LMW HA prepared via chemical and enzymatic degradation has been reported to show a broader MW distribution compared with that prepared using physical techniques [43]. Chemoenzymatic synthesis combines a highly specific enzyme-catalyzed reaction and a flexible chemical reaction, which is useful, especially for the synthesis of non-natural derivatives of HA [14,33].

Another option for in vitro HA synthesis is the enzymatic method. The first cell-free HA synthesis was performed in 1959 using *Streptococcus* membranes, in which the reaction was catalyzed by the membrane-bound enzyme HAS. Isolated HAS can catalyze the same reaction as it does inside the cell in vitro under defined conditions, whereas the final product requires simpler processing and the risk of contamination is reduced. This approach opens more possibilities to control the size of the final product, thus reducing its dispersity [8,46]. Enzymatic catalysis is also a cleaner and greener alternative for the industry, but production costs and the need to improve the stability, activity, and purity of enzymes limit its widespread application [47].

HAS catalyzes the polymerization of HA from its precursor molecules, UDP-GlcA and UDP-GlcNAc, in the presence of divalent metals (e.g., magnesium or manganese),and the preference among scholars is a synthase-dependent process. HAS, specifically its effective kinetic parameters, such as *K_m_* and *V_max_*, significantly influences the MW of HA and its titer. For this reason, HAS protein engineering is a promising tool for the production of HA with desired properties. To identify proper sites to influence HASs, various mutation studies have been conducted, including mutations in membrane regions, cysteine residues, selected conserved residues, upstream and downstream regions of conserved residues, and the C-terminal end of the enzyme [5,48].

## 5. Hyaluronic Acid Synthases

HASs belong to the glycosyltransferase family-2 (GT-2). As noted, HASs require their activity precursors, UDP-GlcA and UDP-GlcNAc, with divalent metals. With their ability to catalyze the polymerization of two UDP–sugars via different glycosidic linkages, they challenge the idea of one glycosyltransferase one type of reaction. However, so far, it appears that HAS transfers only one sugar unit to the nascent chain at a time [49]. Divalent metal coordination is regulated with a conserved DXD motif that is present in many types of GTs. The cation forms a complex with the diphosphate group of the UDP–sugar substrates, facilitating the formation of a transition state and providing assistance in leaving the group [50]. Although HASs were originally categorized into two classes, DeAngelis and Zimmer [49] proposed a new classification based on biochemical and structural work:

1. Class I includes membrane-integrated proteins. As substrates are present in the cytosol, the catalytic domain is cytosolic and functionally integrated with the transmembrane region, which forms a channel. This intrinsic channel is used to secrete the HA directly out of the cell. Class I enzymes are characterized by a processive chain elongation mechanism; i.e., the enzyme retains the HA chain throughout biosynthesis, perhaps because of the close proximity of the GT active site with the channel. These enzymes contain a single GT-2 module that adds both monosaccharide units to the synthesized chain, either to a reducing or non-reducing end. The following are the criteria for subdivision of this class into two subclasses:
  - Class I-NR elongates the HA chain at the non-reducing end and can be found in vertebrates and algal viruses.
  - Class I-R elongates the HA chain at the reducing end and is found in the bacterial genus *Streptococcus*.
2. Class II-NR includes membrane-associated peripheral proteins that require a separate secretion system for HA export. They elongate the chain through a non-processive mechanism. There are two independent GT-2 modules in their structure, one for each type of monosaccharide unit, which are added to the non-reducing end of the synthesized chain. This class is represented by HAS from *P. multocida* (pmHAS) [49,51].

### 5.1. HASs from Vertebrates and the Chlorella algae Virus

**Figure 3:**
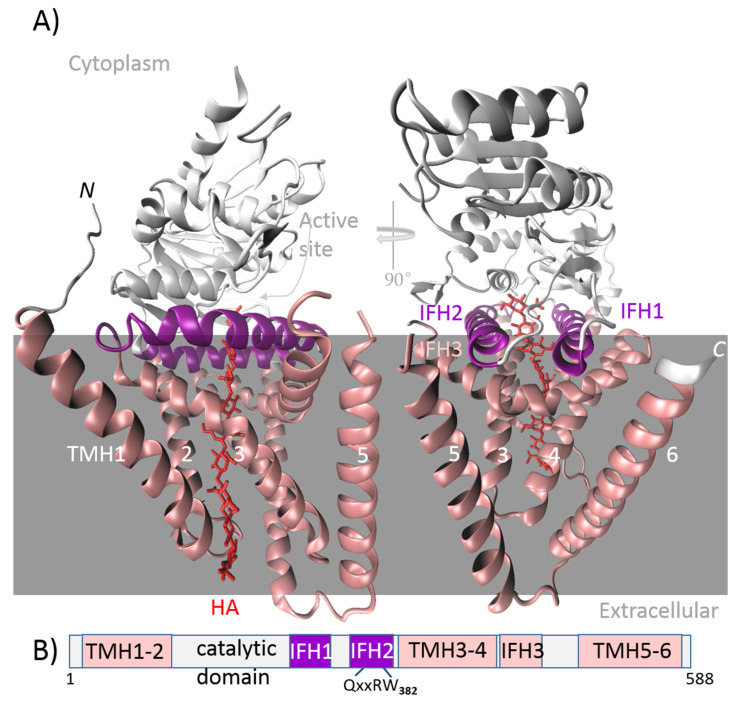
Structure of hyaluronic acid synthase 1 (HAS1) isoform from *Xenopus laevis* (XlHAS1). (**A**) The cytosolic catalytic domain interacts with two N-terminal and four C-terminal transmembrane helices (TMHs) and three cytosolic interface helices (IFHs). XlHAS1’s architecture is consistent with the AlphaFold predicted structure of human HAS1 and HAS2 and the determined cvHAS structure. The coordinates were downloaded from the Protein Data Bank; 8SMN is the corresponding PDB code, and Visual Molecular Dynamics (VMD 1.9.3) was used for visualization. (**B**) Schematic representation of the protein domains of XlHAS1. The conserved QxxRW motif (W382) positions the acceptor sugar moiety directly at the entrance of the transmembrane channel.

Vertebrates have three isoforms of HAS—specifically, HAS1, HAS2, and HAS3—which resemble the viral HAS of PBCV-1 (cvHAS). These enzymes share structural similarities, possessing nine membrane helices. This includes six transmembrane helices (TMHs)—two on the N-terminal and four on the C-terminal side—and three interfacial helices (IFHs) (Figure 3) [49]. In mammals, three isoforms are expressed at specific stages and in specific tissues under normal or pathological conditions. Human HAS1, HAS2, and HAS3 have been successfully expressed and purified using an eukaryotic expression system and tested in vitro. Whereas HAS1 and HAS2 can polymerize HA chains greater than 2 × 10^3^ kDa, HAS3 synthesizes shorter chains with a size between 1 × 10^3^ kDa and 2 × 10^3^ kDa. HAS2 is required at lower concentrations but exhibits a higher rate of HA production compared with the other two isoforms. HAS2 also has a lower *K_m_* for both UDP–sugar precursors than HAS1 and HAS3. These findings are consistent with the idea that HAS2 is dominant among the three isoforms [52], although recombinantly overexpressed human HASs have been observed in the form of homo- and heterooligomers [49]. The HAS1 isoform from *Xenopus laevis* (XlHAS1) was recently biochemically analyzed in vitro and proven to produce HA with a length of ~1600 kDA, comparable to HA synthesized by *Streptococcus dysgalactiae* subsp. *equisimilis* (seHAS) in vitro. On the other hand, under the same conditions, cvHAS produced shorter HA, with a size of ~30–200 kDa. The relatively narrow dispersity of the XlHAS1 HA product broadens after a prolonged in vitro reaction, probably caused by substrate depletion and/or product inhibition. Two substitutions at the entrance to the transmembrane channel, R287K and R296K, abolish length control or terminate HA biosynthesis early, likely due to the disrupted stabilization of the nascent chain for elongation. The mutations K448A or K448R, at about the halfway point of the pore, can reduce the catalytic rate, leading to a shorter yet discrete product. Substitutions that reduce the XlHAS catalytic rate without affecting processivity lead to higher-MW HA [53].

Viral cvHAS contains a typical conserved DXD motif and the second binding site for divalent metals. This second spot is near the binding site for UDP and is important for the catalytic activity of the enzyme, although its role is not yet understood. The substitution of R247 and R256, which are critical for GlcA coordination, resulted in enzyme inactivation, as well as W346 substitution. On the other hand, mutation W248A increased activity by around 20% compared with the wild-type [54]. As previously noted, cvHAS produces in vitro LMW HA chains with relatively broad dispersity. cvHAS and XlHAS function in a monomeric form [53].

### 5.2. Streptococcal HASs

**Figure 4:**
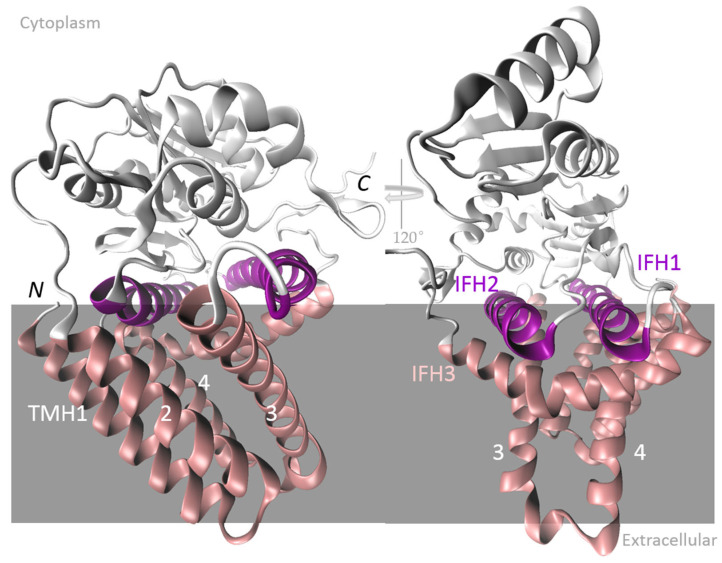
AlphaFold-predicted structure of hyaluronic acid (HAS) from *Streptococcus pyogenes* serotype M18 (spHAS). *Streptococcal* HAS contains two N-terminal and two C-terminal transmembrane helices (TMHs) and three cytosolic interface helices (IFHs). *Streptococcal* HAS probably forms a dimer (not shown), so it lacks two TMHs (TMH5 and TMH6). The coordinates were downloaded from the AlphaFold Protein Structure Database; AF-Q8NKX1-F1-v4 is the corresponding PDB file code, and Visual Molecular Dynamics (VMD 1.9.3) was used for visualization.

As identified in HAS from *S. pyogenes* (spHAS), Streptococcal HASs contain six membrane domains; four of them are integral to forming pores for HA translocation, and two are amphipathic, i.e., membrane-associated regions (Figure 4) [55]. The cytosolic component provides a single function glycosyltransferase domain capable of at least six activities, including two types of UDP–substrate binding (UDP-GlcA; UDP-GlcNAc), two types of HA–sugar binding (HA-GlcA, HA-GlcNAc), and two transferase activities that elongate the nascent chain [48]. The topology of HAS originates from *S. dysgalactiae* subsp. *equisimilis* (seHAS), which revealed that N-terminal residues assist in UDP–sugar binding, while the central domain facilitates substrate binding and glycosyltransferase activity. The hydrophobic part of the C-terminus probably helps to form a channel for the translocation of growing chains across the plasma membrane [56]. C-terminal end deletion has no impact on HA synthesis but is important for HMW HA production, as it stabilizes the enzyme–HA complex [46]. The HAS enzyme has at least two binding sites: one for the UDP–sugar unit and one for polymeric sugar. Results show that there is only one binding site for the two UDP–sugars, suggesting the alternating substrate specificities of these enzymes. Mutation studies have shown that K139 plays a role in substrate binding, probably in stabilizing the polar groups of the substrate, as there is no complete loss of activity when substituted with a charged amino acid. On the other hand, the residues Q295 and T283 have been shown to be crucial for HA biosynthesis [48]. Membrane topology and a tandem motif sequence with two B-X7-B motifs at the seHAS C-terminus are highly conserved in the Class I HAS family. Baggenstoss et al. [57] showed that changes at K398 or within the tandem motif have a reduced HA synthesis rate and HA chain size. Their results suggest that there are two separate HAS functions: one to control HA size and the other to control its synthesis rate [57]. An examination of the truncated form of a HAS enzyme isolated from *S. dysgalactiae* subsp. *equisimilis* Group G (GGS-HAS) showed that, despite a decline in activity, the intracellular domain still performs its binding and polymerization activity without any need for a transmembrane domain [56]. This finding was also confirmed for seHAS by Ebrahimi et al. [46]. Wild-type seHAS showed higher enzyme activity than truncated variants with various deletions in the transmembrane domain, but a variant with a completely deleted transmembrane domain showed significantly higher *K_m_* values for two donor substrates. This lower affinity for the substrate resulted in a reduction in HA’s MW and thus affected the product’s dispersity [46]. Some results suggest that seHAS works as a homodimer [49].

### 5.3. pmHAS

**Figure 5:**
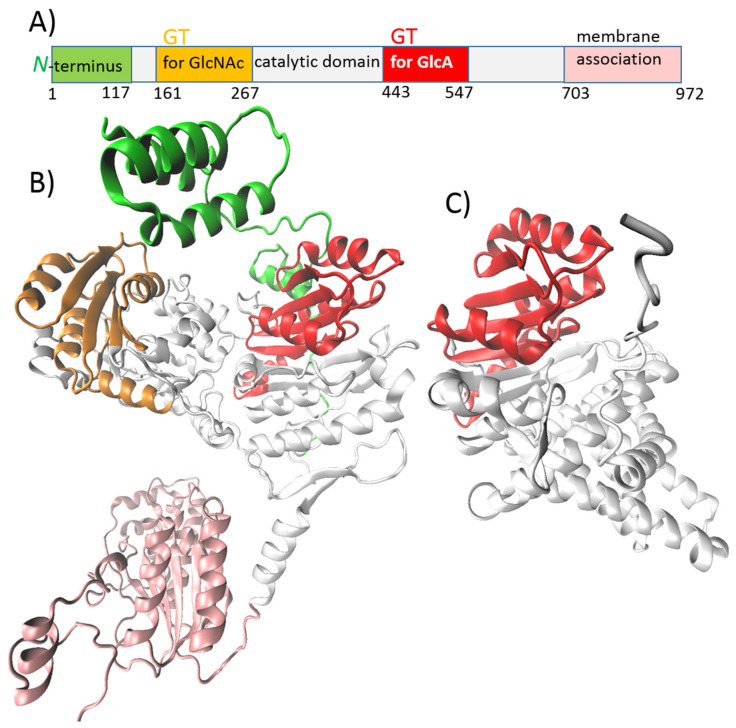
Hyaluronic acid synthase (HAS) from *Pasteurella multocida* (pmHAS). (**A**) Schematic representation of the protein domains of pmHAS. (**B**) AlphaFold-predicted structure of pmHAS. Truncation of residues 1–117 at the N-terminus (green) and the last 269 amino acids at the C-terminus (pink) results in the retention of activity. (**C**) HAS from *Streptococcus pyogenes* serotype M18 (spHAS). Its catalytic domain is sequentially and structurally similar to the pmHAS GT domain for GlcA. The coordinates were downloaded from the AlphaFold Protein Structure Database; AF-Q7BLV3-F1-v4 and AF-Q8NKX1-F1-v4 are the corresponding PDB file codes, and Visual Molecular Dynamics (VMD 1.9.3) was used for visualization.

Class II-NR HAS is represented by pmHAS. Unlike Class I HAS, pmHAS is a peripheral membrane-associated enzyme that acts as a monomer [51]. Jing and DeAngelis [58] prepared a soluble cytoplasmic mutant form of pmHAS (pmHAS^1–703^) by truncating the last 269 amino acids at the C-terminus (Figure 5, pink). These results show that the C-terminal domain is involved in membrane association but does not influence glycosyltransferase activity [51,58]. The truncation of 1–117 residues at the N-terminus led to a similar result, as glycosyltransferase and polymerization activity were retained (Figure 5, green). However, certain point mutations near the N-terminus caused these variants to synthesize HA with a higher MW than the wild-type. It was presumed that these mutations increased the flexibility of the N-terminus [59]. Enzyme pmHAS possesses two independent GT domains, one for GlcNAc (residues 161–267) and one for GlcA (residues 443–547) (Figure 5, orange and red). This was proven using mutation analysis, in which mutations D196N/K/E, D247N/K/E, and D249N/K/E negatively influenced GlcNAc-transferase activity, and mutations D477N/K/E, D527N/K/E, and D529N/K impaired GlcA-transferase activity. The WGGED motif has also been shown to be important for GlcNAc transferase activity. Both GT domains contain the DXD motif, which is involved in metal and UDP–sugar binding, crucial for catalytic activity [58,59,60].

The low sensitivity to inhibition of UDP by-products (~60% inhibited with 15 mM UDP and 1 mM UDP–sugars) makes pmHAS very beneficial for in vitro HA synthesis when compared to Class I HASs (>90% inhibited with UDP levels approaching 0.5 mM) [51]. A non-processive, sequential method of elongation enables the use of pmHAS for the synthesis of defined o-HAs or “quasi-monodisperse” polysaccharides. Stepwise addition reactions are suitable for the synthesis of defined HA in a range of 2 to ~20 monosaccharide units. Short HA tetrasaccharide is used as a primer, and UDP–sugar donors are alternately added to the reaction. Thus, in one step, one UDP-sugar is always added to the nascent chain. However, when using soluble bifunctional pmHAS, the HA oligosaccharide intermediate must be purified before alternative donor addition to avoid a runaway polymerization reaction, which will occur if both UDP–sugar precursors are present simultaneously in a reaction [51]. One can avoid the purification step by using two mutants of pmHAS, each possessing only one transferase activity for GlcA or GlcNAc. This can be achieved by a mutation in the DXD motif. Converting D into N for either of the two DXD motifs does not impact the other catalytic activities, and monofunctional pmHAS is obtained [61].

For the synthesis of longer polysaccharides (~10–2000 kDa), a synchronized, stoichiometrically controlled reaction can be applied to obtain a “quasi-monodisperse” product. This is a one-pot approach that relies on a non-processive mechanism of elongation using pmHAS, in which the ratio of the donor to the acceptor/primer in the reaction will determine the HA chain length. However, pmHAS has a slow initiation phase in vitro, as it cannot efficiently synthesize short HA chains comprising 3–4 monosaccharides. The addition of a short HA oligosaccharide serving as a primer synchronizes the reaction with the product, reaching a polydispersity index of ~1.01 or less (index 1 is the ideal polymer) [51].

The short HA chains used to start these reactions are usually products of the hyaluronidase degradation of HMW HA, containing unsaturated bonds in their molecules and requiring extensive purification. Another option is using a β-1,4-N-acetylglucosaminyltransferase (β1,4EcGnT) derived from the O-antigen gene cluster of *E. coli* O8:K48:H9 for the initial step. β1,4EcGnT exhibits ~12-fold higher GlcNAc transfer activity with the GlcA derivative 4-nitrophenyl-β-D-glucuronide than pmHAS. Disaccharides produced with β1,4EcGnT have been used as a starting material for the stepwise synthesis of HA catalyzed by pmHAS, resulting in a series of HA chains of up to 10 sugar monomers [62].

Various strategies have been applied for various pmHAS immobilization purposes. For example, to develop a visualization technique on a mica surface [63], two monofunctional mutants of pmHAS were immobilized on agarose beads to produce defined HA oligosaccharides [64]; His6-tagged pmHAS was immobilized on metal frameworks to quantify the conversion of HA monomers into disaccharide [65]; and His6-tagged pmHAS was immobilized on NTA-bearing magnetic particles [47] and on nitriloacetic acid functionalized microgels using different bivalent ions via metal binding affinity, both for HMW HA production [66].

### 5.4. One-Pot Synthesis of HA

The nucleotide sugars needed for HA synthesis using HASs are costly substrates. One-pot multi-enzyme synthesis for HA enables the use of cheap basic sugars as primary substrates. The biosynthesis of HA in *S. equi* subsp. *zooepidemicus* starts with glucose that is phosphorylated by glucokinase into glucose-6-phosphate, which is a precursor for both building blocks of HA. For UDP-GlcA synthesis, it is converted by α-phosphoglucomutase (*pgm*) into glucose-1-phosphate, which serves as a substrate for UDP–glucose phosphorylase (*hasC*) to produce UDP–glucose. UDP–glucose is then oxidized by UDP–glucose dehydrogenase (*hasB*) into UDP-GlcA. For UDP-GlcNAc synthesis, glucose-6-phosphate is converted by phosphoglucoisomerase (*hasE*) into fructose-6-phosphate which, after tagging with an amido group, is converted by amidotransferase (*glmS*) into glucosamine-6-phosphate and modified by mutase (*glmM*) into glucosamine-1-phosphate. The acetylation and phosphorylation of this intermediate using acetyltransferase and pyrophosphorylase (*hasD*) then produces UDP-GlcNAc. UDP-GlcA and UDP-GlcNAc are then polymerized by HAS (*hasA*) [8].

One-pot reactions for HA production include two enzyme modules for nucleotide sugar synthesis and one module for HA polymerization. One-pot synthesis with in situ nucleotide sugar regeneration starts with sugar-1-phosphates, using HAS-containing membrane preparations of *S. pyogenes* and six other enzymes. This led to the production of HA with an MW of 550 kDa [67]. Eisele et al. [10] used sucrose and GlcNAc as starting substrates. Potato-derived sucrose synthase generates UDP–glucose from sucrose and UDP, and UDP–glucose dehydrogenase from *S. equi* subsp. *zooepidemicus* generates UDP-GlcA from UDP–glucose; this reaction is coupled with NADH oxidase for NAD^+^ regeneration. GlcNAc is phosphorylated by N-acetylhexosamine-1-kinase from *Bifidobacterium longum* and then GlcNAc-1-phosphate is converted into UDP-GlcNAc by GlcNAc-1-phosphate uridyltransferase from *S. equi* subsp. *zooepidemicus*. Pyrophosphatase is used to drive this reaction to UDP–sugars. The final step of HA production is catalysis with pmHAS. The by-product of this reaction is UDP, which is regenerated in situ for nucleotide sugar synthesis. Using this reaction scheme, HA with an MW > 2000 kDa was synthesized [10].

**Figure 6:**
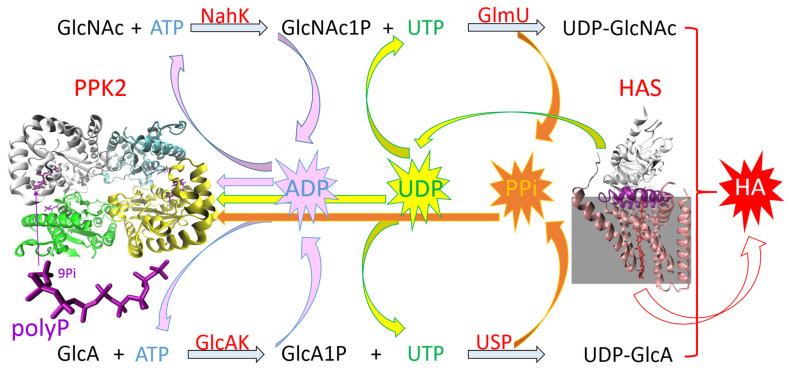
The most promising one-pot synthesis system for hyaluronic acid (HA). NahK—N-acetylhexosamine-1-kinase; GlmU—GlcNAc-1-phosphate uridyltransferase; GlcAK—GlcA-1-kinase; USP—UDP-sugar pyrophosphorylase; PPK2—polyphosphate kinase class 2; and HAS—hyaluronic acid synthase. Different PPK2s and HASs with different preferences for polyP/NDP and differing performance in HA synthesis can be used. The tetrameric structure of PPK2 from *Francisella tularensis* SCHU S4 with polyphosphate and the structure of the HAS1 isoform from *Xenopus laevis* are shown. The coordinates were downloaded from the Protein Data Bank; 5LL0 and 8SMN are the corresponding PDB codes.

Another approach uses GlcA and GlcNAc as primary substrates (Figure 6). GlcA is converted into UDP-GlcA by GlcA-1-kinase and UDP–sugar pyrophosphorylase, both from *Arabidopsis thaliana*. The module for UDP-GlcNAc resembles the one described above, except that GlcNAc-1-phosphate uridyltransferase originates from *Campylobacter jejuni*. HA is polymerized by pmHAS in a stepwise fashion. In a preparative scale reaction system, the yield reaches over 70%, and HA’s MW ranges from 15 to 550 kDa. This method has also been used for the incorporation of unnatural monosaccharide analogs [68].

#### ATP/UTP Regeneration System

UTP and ATP are required as energy sources. UTP activates sugar substrates, and ATP is required for the phosphorylation of intermediates [69]. Regenerating UTP and ATP (NTP) in cell-free systems, in which nucleotides are not metabolically replenished, is crucial for efficient and cost-effective synthesis. Several kinase/phosphate donor recycling systems are commonly used in NTP regeneration [70]. One of the most commonly used is a system using acetate kinase and acetyl phosphate as a phosphate (Pi) donor. However, it has several disadvantages that may limit its use. The released acetate can lower the pH and potentially affect enzyme activity. Acetyl phosphate is relatively unstable, which may reduce the efficiency of ATP regeneration or affect product purity [71]. Another well-established method used in ATP regeneration is the phosphoenolpyruvate (PEP) regeneration system. PEP is a high-energy phosphate donor and, in the presence of pyruvate kinase, it can efficiently regenerate ATP from ADP. The PEP system has several advantages, including a high energy yield, compatibility with HA enzymes, and rapid turnover, all of which support long-term production. On the other hand, several disadvantages need to be considered, such as the cost of PEP, pyruvate accumulation, and buffering (pyruvate and phosphate can acidify the reaction) [70,71]. Creatine kinase with creatine phosphate (CP) represents an alternative to acetate kinase and pyruvate kinase, with the main advantage of CP being the price [71].

In recent years, the polyphosphate–polyphosphate kinase (polyP/PPK) system has emerged as a promising, cost-effective, and scalable technique. It uses polyP as a phosphate donor and PPK to phosphorylate nucleotide diphosphate (NDP) into NTP (Figure 6). PolyPs are linear polymers composed of a variable number of phosphate residues linked by high-energy phosphoanhydride bonds. They are evolutionarily ancient biopolymers that occur from bacteria to humans, playing a significant role in many biological processes—from energy and phosphate storage and regulatory processes to influencing cellular metabolism, growth, and differentiation. No other molecule concentrates as much (bio)chemically usable energy [72]. In 2009, Nahálka and Pätoprstý identified the first PPK (from *Ruegeria pomeroyi*) that prefers pyrimidine over purine NDP but is also capable of accepting both [73]. It is called PPK3 because PPKs were originally divided into PPK1 (which preferentially uses ATP/GTP to synthesize polyP) and PPK2 (which preferentially phosphorylates ADP/GDP using polyP) [72]. At present, PPK2 enzymes are divided into three subclasses: PPK2-I (which phosphorylates NDP); PPK2-II (which phosphorylates nucleotide monophosphate (NMP)); and PPK2-III (which phosphorylates both NMP and NDP) [74]. In 2021, Gottschalk et al. presented a significant advance in the enzymatic synthesis of HA when they integrated *Ruegeria pomeroyi* PPK3 (RpPPK2-3) into the system [75]. In their subsequent work, they improved the operational stability, recyclability, and economic feasibility by implementing His-Tag-based immobilization in this system. Compared with the soluble enzyme, they achieved a much higher average molecular weight of HA (3600 kDa) and a comparable HA concentration after the first cycle (368 vs. 410 mg·L^−1^). However, the system had limitations, and they observed a significant decrease in enzymatic activity (especially pmHAS) after the first cycle [47]. Zhen Kang and colleagues developed a robust and reusable enzymatic system for the synthesis of both HAS substrates; furthermore, to recycle the accumulated ADP and pyrophosphate (PPi), they introduced SePPK from *Staphylococcus epidermidis* into the system, which significantly increased the yields of UDP-GlcNAc and UDP-GlcA [76]. Previously, they found that some PPKs, such as SePPK, are capable of accepting PPi to regenerate ATP (Figure 6) [77].

The abovementioned results suggest that the polyP/PPK regeneration system may offer a robust solution for efficient, large-scale, and continuous HA production. By enabling in situ NTP regeneration using inexpensive and stable polyP, the dependence on costly cofactors is reduced, and the sustainability of the process is increased.

## 6. Application of HA

**Figure 7:**
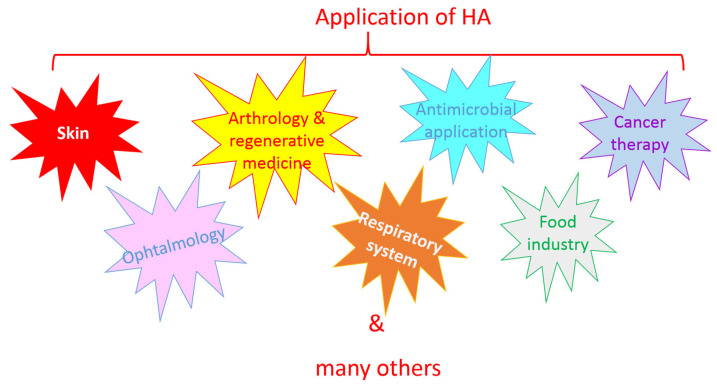
Hyaluronic acid (HA) is an ideal molecule for application in various fields.

As a naturally occurring polysaccharide, HA is biocompatible, non-immunogenic, biodegradable, and available for various modifications, making it an ideal molecule for application in various fields, including cosmetics, medicine, and the food industry (Figure 7).

### 6.1. Skin

In the field of cosmetics, HA is used as a component of various products. Due to its biological properties, it shows promising efficacy in skin hydration, anti-aging and anti-wrinkle action, face rejuvenation, skin augmentation, collagen stimulation, and improving the skin’s elasticity and tightness [12,15]. The hydrating effect of HA varies according to its MW. HMW HA cannot penetrate the deeper skin layers, remaining instead in the stratum corneum where it forms a breathable film on the skin surface and provides long-term moisturization. LMW HA exhibits better permeability in the epidermis and dermis; thus, it is able to moisturize deeper layers. Penetration might be enhanced with electroporation, iontophoresis, or microneedles. HA with a molecular weight of <10 kDA can penetrate into the dermis and regulate skin metabolism [78]. There is also increasing interest in the use of HA in esthetic medicine as a tissue filler for soft tissue augmentation, nasolabial fold and wrinkle correction, and lip enhancement. For this purpose, cross-linked HA is the most efficient, as non-cross-linked HA has a short half-life [79,80]. Besides the passive improvement of the skin through its physico-chemical properties, various preparations of HA also enhance collagen synthesis, contribute to the formation of new extracellular matrix, and modulate the expression of genes related to skin moisturization [79,81,82].

Apart from cosmetic and esthetic areas, HA also has medical applications in skin. HA impacts cell activation, inflammatory responses, and wound repair processes. Wound healing is a complex process including inflammatory responses, cell proliferation, collagen deposition, fibrous tissue formation, angiogenesis, and scar shaping. HMW HA, LMW HA, and HA oligosaccharides are involved in various stages of wound healing [15,83]. HA in the form of hydrogels, sponges, films, creams, or foams—often in combination with other substances—has proven efficiency in healing various ulcers and wounds, including difficult and slow-healing diabetic wounds [83,84,85]. Evidence suggests that the use of HA is a potential strategy for the treatment of inflammatory skin diseases, such as rosacea [86].

### 6.2. Ophthalmology

The moisturizing, anti-inflammatory, and healing-supporting properties of HA are also employed in ophthalmology. HA can be used in eyedrops or in situ to form a hydrogel that can be applied to lens embedding, dry-eye treatments, ulcerative keratitis, or as a supporting treatment associated with ophthalmic surgeries, such as cataract extraction or vitreous retinal operations [87,88]. HA hydrogel and nanoparticles can be used as drug delivery systems for various eye diseases [89,90,91].

### 6.3. Arthrology and Regenerative Medicine

HA is an important component of articular cartilage tissue and synovial fluid. A decrease in the concentration and MW of HA can result in reduced viscoelasticity, which is typical in osteoarthritis and rheumatoid arthritis. However, in the latter, it is associated with the overexpression of certain immune cells with various cell surface receptors, such as CD44. Thus, there is a particular interest in HMW and cross-linked HA for the development of HA-based systems to deliver various substances and manage arthritis. Various sources show that intraarticular injection with HA stimulates the synthesis of articular cartilage matrix components, promotes healing and cartilage repair, and inhibits inflammatory processes [92,93,94,95].

During the treatment of neurodegenerative diseases and neural tissue injuries, various biomaterials have been injected in the form of hydrogels to fill irregular injury sites, deliver or sequester various molecules, or impact neural cells. HA is a promising material for regeneration therapy in neurology, as it can facilitate a supportive and biomimetic environment [96,97]. Cross-linked HA hydrogels, often combined with collagen or other substances, are capable of facilitating the localized, long-term delivery of neurotrophic factors and certain neuroprotective agents, thus promoting the stabilization and regeneration of axons [98,99]. HA-derived scaffolds prepared using electrospinning or 3D bioprinting show potential in creating ideal microenvironments for neural tissue regeneration, which may even be suitable for stem cell therapy. This could facilitate cellular migration and proliferation and the survival of embedded stem cells [97,100]. Scaffolds based on HA are also applicable in the engineering other tissues, such as skeletal muscle, skin, or dental regeneration [100].

HA has been proven to have a positive effect on osteogenesis. Hydroxyapatite and β-calcium triphosphate, combined with a mix of HMW and LMW HA, can improve the operability of granular bone grafts and enhance new bone formation. Whereas HMW HA provides a scaffold, LMW HA has a positive effect on cell proliferation and differentiation, and influences the inflammatory response. An HMW/LMW HA ratio of 50:50 has been shown to be the most plausible, as it exhibits acceptable viscosity and significant new bone formation [101]. Similar results have also been obtained for guided bone regeneration surgery in dentistry. LMW HA with carboxymethyl cellulose (CMC) showed better bone-healing properties than an HMW HA–CMC hybrid. LMW HA can bind CD44 on the dental pulp stem cell membranes and affect the mitogen-activated protein kinase pathway, increasing the proliferation rate [102]. The LMW HA used in the abovementioned research was prepared using γ-irradiation [101,102].

### 6.4. Respiratory System

HA is also a naturally occurring compound in the lungs and respiratory tissue. HMW HA has been added to decongestants, seawater nasal sprays, and inhalatory solutions as an airway hydrating agent. This may improve mucociliary transport, ameliorate airway hyper-responsiveness, provide an anti-inflammatory effect, and reduce lung epithelial injury. HA has been applied to patients suffering from rhinitis and rhinosinusitis, nasal polyposis, empty nose syndrome, or as a part of postoperative care after nasal surgery. It also shows positive effects in patients with chronic obstructive pulmonary disease, asthma, and cystic fibrosis [103,104,105]. Moreover, HA might serve as a carrier for other treatment substances, improving their stability and ability to reach the deep lung. For example, HA–vancomycin complex powder can be used to treat pulmonary infections in patients with cystic fibrosis, or HA–dexamethasone nanoparticles can be used in lung inflammation therapy, both via inhalation [106,107]. Subcutaneously injectable HA hydrogel containing house dust mite was proven to be an efficient immunotherapy that can reduce allergic symptoms and induce tolerance in a murine model [108].

### 6.5. Antimicrobial Application

HA has potential applications in medicine due to its antimicrobial activity. Not only does HA act as a carrier of antimicrobial compounds, but it also has microbistatic effects. It has been proven to be efficient against various Gram-positive (e.g., *Staphylococcus aureus* and β-hemolytic *Streptococcus*), Gram-negative bacteria (e.g., *Pseudomonas aeruginosa* and uropathogenic *E. coli*), some fungal strains (e.g., certain strains of *Candida albicans*), and some viruses (e.g., HIV, mumps virus, and influenza virus A/H1N1). Many bacteria produce HA-degrading enzymes (HA lyases) as a virulence factor to promote invasiveness and the local spread of infection. Exogenous HA might be able to overwhelm HA lyases, thereby suppressing bacterial growth. Moreover, due to its hydrophilicity and negative net charge, HA inhibits biofouling and biofilm formation. The antiviral mechanism of HA probably involves the inhibition of viral activity by binding with the envelope ligand; inhibition of viral docking, internalization, and uncoating in the host cell; and improving the immune response of the host. Although all MWs of HA exhibit antimicrobial effects at some level, HMW HA is more effective than LMW HA [27]. To improve mechanical properties and stability, cross-linked HA and its derivatives can be used as coatings for various implantable devices, catheters, prostheses, etc., where biofilm-related infections are major problems [109].

### 6.6. Cancer Therapy

HA-based nanocarriers are a promising tool in cancer therapy for improving solubility, stability, cytotoxicity, and the insufficient tumor uptake of traditional anticancer drugs. Moreover, they can carry natural products with anticancer activity, compounds for gene therapy, and oncolytic viruses [110,111]. The CD44 receptor of HA is overexpressed on tumor cells. Thus, HA nanostructures are suitable for targeted therapy, promoting cellular uptake and internalization via HA-specific receptor-mediated endocytosis [110,111,112,113]. Some types of HA nanocarriers are seemingly capable of sustained drug release [112,114]. The biodegradable scaffold of these particles is usually made of LMW HA [112,115], but HA tetrasaccharide conjugated with an anticancer agent with a disulfide linkage can also improve solubility, exhibiting significant antitumor efficacy [113]. HA hydrogels can be used in an injectable form as spacers during radiotherapy to reduce radiation-associated toxic side effects by creating physical distance between the target and normal tissue [110].

### 6.7. Food Industry

An emerging field of application for HA is the food industry. Several countries have approved HA as a functional food and food additive, and it can now be found in various foods, such as yogurts, fruit juices, and soybean milk. The beneficial effects of orally administered HA have also been reported, e.g., for the skin and knee osteoarthritis, and it has even demonstrated a prebiotic effect. HA can also serve as a natural seasoning agent and taste enhancer. Moreover, it shows potential as a versatile antibacterial, non-toxic, and biodegradable food packaging material [116].

## 7. Conclusions and Future Directions

HA is a biomolecule with a variety of functions in the human body, as well as in other vertebrates. Its natural characteristics make it the center of numerous studies, such as its biodegradability, biocompatibility, mucoadhesivity, hygroscopicity, and viscoelasticity, as well as its ability to undergo various modifications. HA is widely used in cosmetic products, esthetic medicine, and other fields of medicine, e.g., wound healing, ophthalmology, arthrology, and pneumology. It also shows great potential for use in the therapeutic treatment of cancer and other diseases, as a nanocarrier capable of specific targeting and long-lasting drug delivery. It has been proven to be a suitable material for scaffolds in regenerative medicine and for various food industry implementations. HA is a very dynamic molecule, occurring in many length variants, with its MW being a crucial determinant of its activity in various physiological or pathological processes. Therefore, the practical application of HA requires a full understanding of its biological roles in the human body at different sizes. The use of tailored-MW HA could improve its desired effects and prevent unwanted influence.

The production of HA of a defined length is a significant challenge. The oldest approach is extraction from animal sources, which results in a highly polydisperse product. Microbial fermentation is currently the dominant method, providing limited options for regulation of the MW of the final product, although research in this area is still ongoing. Besides polydispersity, extraction and fermentation require rigorous purification to account for safety concerns. Enzymatic HA synthesis in vitro presents good possibilities for regulation and monodisperse HA synthesis under defined conditions. A crucial aspect of successful in vitro synthesis is research on HASs and their protein engineering. This could help to increase yields and/or restrict product dispersity, as these enzymes not only catalyze the polymerization of monomeric units but also regulate the MW of the nascent chain. The associated regulation mechanism is not yet fully understood. To date, methods for the in vitro synthesis of monodisperse or “quasi-monodisperse” HA have used pmHAS [51]. However, research on HASs from the bacterial genus *Streptococcus*, vertebrates, and PBCV-1 is also in progress [46,53,54].

Large-scale synthesis also requires cheap substrates. The substrates used for HASs—namely, UDP-GlcA and UDP-GlcNAc—are costly. Therefore, HASs must be coupled with other enzymes for the activation of cheap basic sugars and NTP regeneration systems. NTP regeneration systems are a key aspect of efficient HA synthesis. Although there are several possibilities, polyP is a promising source of chemically usable energy and, in combination with the enzyme PPK, it seems to be plausible for this purpose. It has been successfully applied in a one-pot multi-enzyme reaction for the synthesis of HMW HA, although further optimization of the system is necessary [47]. The polyP/PPK regeneration system is a promising solution for the efficient one-pot enzymatic production of HA.

## Abbreviations

CD44	Cluster of differentiation 44
CP	Creatine phosphate
GlcA	D-glucuronic acid
GlcNAc	N-acetyl-D-glucosamine
GT	Glycosyltransferase
HA	Hyaluronic acid
HARE	Hyaluronan receptor for endocytosis
HAS	Hyaluronic acid synthase
HMW HA	High-molecular-weight hyaluronic acid
IFH	Interfacial helix
LMW HA	Low-molecular-weight hyaluronic acid
LYVE1	Lymphatic vessel endothelial hyaluronan receptor 1
MW	Molecular weight
NDP	Nucleotide diphosphate
NTP	Nucleotide triphosphate
o-HAs	Oligosaccharides of hyaluronic acid
PEP	Phosphoenolpyruvate
Pi	Phosphate
polyP	Polyphosphate
PPi	Pyrophosphate
PPK	Polyphosphate kinase
RMAHH	Receptor for HA-mediated cell motility
TLR	Toll-like receptor
TMH	Transmembrane helix
vHMW HA	Very-high-molecular-weight hyaluronic acid
